# The influence of etiology on surgical outcomes in neovascular glaucoma

**DOI:** 10.1186/s12886-021-02212-x

**Published:** 2021-12-20

**Authors:** Charles M. Medert, Catherine Q. Sun, Elizabeth Vanner, Richard K. Parrish, Sarah R. Wellik

**Affiliations:** 1 Bascom Palmer Eye Institute, University of Miami Miller School of Medicine, 900 NW 17th Street, Miami, FL 33136 USA; 2 University of California San Francisco, San Francisco, CA USA

**Keywords:** Glaucoma, Neovascular, Surgery, Diabetes, Vision, Outcomes

## Abstract

**Background:**

The authors sought to evaluate visual outcomes in patients with varying etiologies of neovascular glaucoma (NVG), who were treated with glaucoma drainage devices (GDD).

**Methods:**

This was a retrospective case series of patients at a large academic teaching institution who had surgical intervention for neovascular glaucoma between September 2011 and May 2019. Eyes were included if there was documented neovascularization of the iris/angle with an intraocular pressure (IOP) > 21 mmHg at presentation. Eyes must also have been treated with surgical intervention that included a GDD. Primary outcome measure was visual acuity at the 1-year post-operative visit. Secondary outcome measure was qualified success after surgery defined by: pressure criteria (5 mmHg < IOP ≤ 21 mmHg), no re-operation for elevated IOP, and no loss of LP vision.

**Results:**

One hundred twenty eyes met inclusion criteria. 61.7% had an etiology of proliferative diabetic retinopathy (PDR), 23.3% had retinal vein occlusions (RVO), and the remaining 15.0% suffered from other etiologies. Of patients treated with GDD, eyes with PDR had better vision compared to eyes with RVO at final evaluation (*p* = 0.041). There was a statistically significant difference (*p* = 0.027) in the mean number of glaucoma medications with Ahmed eyes (*n* = 70) requiring 1.9 medications and Baerveldt eyes (*n* = 46) requiring 1.3 medications at final evaluation.

**Conclusions:**

In our study, many patients with NVG achieved meaningful vision, as defined by World Health Organization (WHO) guidelines, and IOP control after GDD. Outcomes differed between patients with PDR and RVO in favor of the PDR group. Different GDD devices had similar performance profiles for VA and IOP outcomes. Direct prospective comparison of Baerveldt, Ahmed, and cyclophotocoagulation represents the next phase of discovery.

## Background

Ocular ischemic diseases often cause severe vision loss from neovascular glaucoma (NVG) [1]. Some of the most common causes of neovascularization are proliferative diabetic retinopathy (PDR) and ischemic central retinal vein occlusion (RVO), but other causes include ocular ischemic syndrome and inflammatory disorders [1]. Neovascularization itself is thought to be caused by a variety of angiogenic factors that are triggered by the initial retinal ischemia [2]. Ultimately there is neovascularization of the anterior chamber angle leading to trabecular meshwork dysfunction and intractable, elevated intraocular pressure (IOP) [2]. Patients with NVG often present abruptly, may be challenging to manage, and may need urgent surgical management for control of elevated IOP, particularly once peripheral anterior synechiae form [3]. Even with ideal management given in a timely fashion, NVG can result in a poor visual prognosis [3].

The prevalence of NVG is estimated at 52 per 100,000 in a recent nationwide population based database study in Taiwan with an observed increase in the percentage of patients receiving anti-vascular endothelial growth factor (anti-VEGF) treatments and a decrease in the incidence of trabeculectomy for treatment over the study period [4]. In addition, diabetic retinopathy, one of the leading causes of NVG, was found to be the 5th most frequent cause of blindness in those aged 50 years and older in 2020 according to a recent analysis for the Global Burden of Disease Study [5]. Historically, management of NVG has included treatment of the retinal neovascularization with laser and anti-VEGF treatments [1, 6] and control of IOP through medical and surgical intervention [7]. High rates of failure with conventional trabeculectomy surgery [8–10] have led to increased use of GDD for treatment of NVG [7, 10, 11]. Vision outcomes are thought to be poor for NVG [12], but most studies have used a limited definition of success including not progressing to no light perception (NLP) vision [13, 14].

To our knowledge, there have been no randomized clinical trials, and only a few prospective [15, 16] and retrospective studies [ 14, 17, 18], evaluating the outcomes of glaucoma surgery for NVG. The large prospective AVB and ABC trials were not designed with neovascular glaucoma specifically in mind but rather performed sub analyses of the small proportion of NVG patients enrolled and had a limited definition of surgical success [19–21]. A more recent retrospective analysis of NVG surgery by Shalaby et al. analyzed valved versus non-valved GDD for NVG but did not look at NVG etiology in the surgical outcomes and also used the same definition of surgical success as the prior prospective trials [14].

No previous reports, that we are aware of, specifically address the differences in surgical outcome between etiologies of NVG, such as the differences in surgical outcomes between PDR and RVO patients. We know that many of these patients ultimately have vision worse than 20/400 and some progress to light perception (LP) or NLP vision. This study aims to analyze prognostic factors that would be useful for individualizing treatment based on presenting factors, including etiology of NVG. This would be helpful for educating patients during the informed consent process.

With an aim to close these knowledge gaps, we reviewed the medical records of NVG patients treated with GDD to determine the outcomes in patients with PDR, RVO, and other etiologies 1 year after glaucoma surgery. Our main outcome measure is the average visual acuity (VA) between different etiologies of NVG. Secondarily, we analyzed traditional surgical success as defined in other studies [22]. In addition, we give the change in VA between surgery and 1 year follow up, which we do not believe any other publications have done. Finally, we present an analysis on presentation characteristics that may be associated with better (20/400 or better) and worse (hand motion or worse) VA outcomes at post-operative year 1 after GDD surgery for NVG.

## Methods

This was a retrospective, single center, comparative cohort study. The University of Miami Clinical and Translational Science Institute performed an electronic review of data from our hospital electronic medical record (EMR) (EPIC Systems Corporation, Verona WI) to identify all patients with a diagnosis of NVG presenting to the Bascom Palmer Eye Institute / Anne Bates Leach Eye Hospital (emergency department and outpatients visits) between 9/1/2011 through 5/1/19 with an ICD-9 or ICD-10 diagnosis of neovascular glaucoma (we used ICD9 365.63, Glaucoma associated with vascular disorders and ICD10 H40.89, other specified glaucoma). We then used the same system to identify all patients for the same time period with CPT codes for GDD surgery (aqueous shunt with and without graft - 66,179, 66,180). These records were then cross-referenced in Excel 2020 (Microsoft Corporation, Redmond, WA) and manually reviewed to check for inclusion/exclusion criteria. This retrospective review was performed independently for this study and did not have overlap with any other study at our institution. Inclusion criteria were: neovascularization of the iris or angle documented in the clinical notes, and an IOP > 21 mmHg at presentation. Exclusion criteria were age < 18 years, history of the previous glaucoma procedures (cyclodestructive surgery, trabeculectomy, glaucoma drainage implant) in the presenting eye, and no 3-month follow-up visit after surgery. The Institutional Review Board at the University of Miami School of Medicine approved this protocol before beginning the study and it followed the tenets of the Declaration of Helsinki.

For the baseline visit, we used the visit at which neovascularization was first noted, which we refer to as the presentation visit. We recorded any treatments the patients had had (whether at our institution or a referring provider) including anti-Vascular Endothelial Growth Factor (anti-VEGF) injection, pan-retinal photocoagulation (PRP), and topical and systemic glaucoma medications. Records of slit lamp examination were used to assess documentation of neovascularization of the iris, angle or posterior pole and lens status. Best corrected and/or pinhole Snellen VA, IOP taken by either Goldman applanation tonometry or TonoPen (Reichert Technologies, Depew, New York), and glaucoma medications (prostaglandin analog (PGA), beta blocker, alpha agonist, miotic, topical carbonic anhydrase inhibitor, oral carbonic anhydrase inhibitor) were recorded at presentation, and at approximate post-operative day 1, month 3, and 1 year visits. For month 3, accepted range was 2–5 months, and for 1 year the accepted range was 7–26 months. Post-operative complications were also recorded. All subsequent re-operations in the study eye were also recorded during the study period. Analyses of final values (IOP, VA, and number of glaucoma medications) used data from the last data point available.

The primary outcome measure for this study was the final visual acuity. Snellen VA were converted to logMAR for analysis, and non-Snellen visual acuities were converted as follows: count fingers = 2.0, hand motions = 2.3, light perception = 2.6, and no light perception = 3.0. The secondary outcome measure was qualified success at the final evaluation after surgery defined by IOP criteria (5 mmHg ≥ IOP ≤ 21 mmHg, with or without IOP lowering medication), no re-operation for elevated IOP at 1 year, and no loss of LP vision at 1 year. Additionally, we assessed for factors associated with functionally better vision as defined by 20/400 or better and functionally poor vision as defined by hand motion or worse vision.

### Statistical analysis

Categorical variables were compared with chi-square, Fisher exact, and exact chi-square tests and ordinal variables were compared with the Jonckheere-Terpstra test; both are presented as frequencies and percentages. Continuous variables were compared with the Mann-Whitney Wilcoxon and Kruskal-Wallis tests and are presented as means and standard deviations (SD). Treatment groups were compared on final visual acuity and the change in final visual acuity from presentation using least-squares regression that accounted for the correlation between both eyes of a subject. Treatment groups were compared on the secondary composite outcome measure and for whether they had functionally better or functionally poor vision as defined above, using logistic regression that accounted for the correlation between both eyes of a subject. All analyses were done using SAS version 9.4 (SAS Institute, Cary, North Carolina, USA). A *P* value < 0.05 was considered statistically significant.

## Results

One thousand eight hundred fifty eyes were identified with a diagnosis of NVG based on diagnostic codes. After cross referencing for those who had glaucoma surgery and applying inclusion and exclusion criteria, 120 eyes remained. 113 eyes had a single GDD placed at the time of surgery, while 4 eyes received two tubes and 3 eyes received a GDD with an orphan trabeculectomy. Inaccurate coding and/or lack of documented neovascularization were the most frequent reason for exclusion. Patients were predominantly Caucasian (46.9%) or African American (46.9%), non-Hispanic (56.6%), and Male (59.3%) in the single GDD group. Of the 120 eyes that met inclusion criteria, 109 eyes were followed through approximately 1 year. We present the data points as 3 months (mean 3.3) and final (mean 12.4) with 3-month data being used as surrogate final VA and IOP for those few patients (*n* = 11) missing 1-year data. A sub-analysis is presented later in this section showing that this did not change statistical outcomes.

The etiology of neovascularization was broken down into 3 groups. Of the 120 eyes, most (61.7%, *n* = 74) presented with NVG secondary to proliferative diabetic retinopathy. The second most frequent etiology (23.3%, *n* = 28) was retinal vein occlusion. Vein occlusions were reported as central retinal vein occlusion (78.6%, *n* = 22), branch retinal vein occlusion (17.9%, *n* = 5), and hemi-retinal vein occlusion (3.6%, *n* = 1). The remaining “Other Etiology” group (15.0%, *n* = 18) were caused by: ocular ischemic syndrome (OIS), central retinal artery occlusion (CRAO), and ischemia related to intra-ocular tumors, uveitis, or retinal detachment/detachment repair. The breakdown of type of surgical intervention and etiology of NVG is presented in Table 1. Demographic characteristics for GDD eyes are summarized in Table 2. Of note, the only significant demographic difference on presentation between the various etiologies was that RVO and Other Etiology patients were more likely to be older on presentation than PDR patients (Table 3).

**Table 1:** Surgical characteristics of neovascular glaucoma patients (*n* = 120)

	ALL	PDR	RVO	Other
GDD				
Baerveldt (BGI)	46	29 (63%)	10 (22%)	7 (15%)
Ahmed (AGV)	70	43 (61%)	17 (24%)	10 (14%)
AGV + BGI	4	2 (50%)	1 (25%)	1 (25%)
GDD (BGI) + Orphan Trabeculectomy	3	2 (66%)	0 (0%)	1 (44%)
GDD (BGI) + CE/IOL	4	3 (75%)	1 (25%)	0 (0%)
GDD (AGV) + CE/IOL	4	3 (75%)	1 (25%)	0 (0%)
GDD + Retinal Surgery	3	1 (33%)	1 (33%)	1 (33%)
Tube location				
Anterior Chamber	103	64 (62%)	26 (25%)	13 (13%)
Sulcus	13	8 (62%)	2 (15%)	3 (23%)
Pars plana	4	2 (50%)	0 (0%)	2 (50%)
Panretinal photocoagulation at surgery	10	7 (70%)	1 (10%)	2 (20%)
Intravitreal injection at surgery (VEGF or Steroid)	10	8 (80%)	1 (10%)	1 (10%)

**Table 2:** Demographic characteristics for patients who were treated with glaucoma drainage devices

	All		PDR		RVO		Other	
	n	%	n	%	n	%	n	%
Sex								
Male	73	60.83	46	(62.2%)	16	(57.1%)	11	(61.1%)
Female	47	39.17	28	(37.8%)	12	(42.9%)	7	(38.9%)
Race								
Black	56	46.67	39	(52.7%)	10	(35.7%)	7	(38.9%)
White	57	47.50	32	(43.2%)	15	(53.6%)	10	(55.6%)
Unknown	2	1.67	1	(1.4%)	0	(0%)	1	(5.6%)
More than one	5	4.17	2	(2.7%)	3	(10.7%)	0	(0%)
Ethnicity								
Non-Hispanic	66	55	42	(56.8%)	15	(53.6%)	9	(50%)
Hispanic	54	45	32	(43.2%)	13	(46.4%)	9	(50%)
Hx of Hypertension								
Yes	104	87.39 (*n* = 119)	67	(91.8%)	24	(85.7%)	13	(72.2%)
Hx of Diabetes mellitus								
Yes	101	84.17 (*n* = 120)	74	(100%)	16	(57.1%)	11	(61.1%)
Type 1	2	2	1	(1.4%)	1	(6.3%)	0	(0%)
Type 2	98	98	72	(98.6%)	15	(93.7%)	11	(100%)

**Table 3:** Ophthalmic history: NVG patients treated with a glaucoma GDD

	ALL	n	PDR	n	RVO	n	Other	n
VA at presentation (Log Mar)	1.61 (SD: 0.73)	120	1.66 (SD: 0.73)	74	1.45 (SD: 0.75)	28	1.68 (SD: 0.71)	18
IOP at presentation (mmHg)	43.7 (SD: 13.5)	120	44.5 (SD:13.7)	74	42.4 (SD: 10.5)	28	42.5 (SD: 16.9)	18
Age at presentation (years)^a^	66.9 (SD: 11.7)	120	58.2 (SD: 8.8)	74	72.4 (SD: 11.7)	28	65.4 (SD: 12.4)	18
Non-neovascular Glaucoma before presentation	30 (25%)	119	13 (17.6%)	73	10 (35.7%)	28	7 (38.9%)	18
Lens Status at presentation								
Phakic	53 (48%)	110	36 (52.9%)	68	11 (45.8%)	24	6 (33.3%)	18
Pseudophakic	55 (50%)	110	31 (45.6%)	68	13 (54.2%)	24	11 (61.1%)	18
Aphakic	2 (1.8%)	110	1 (1.5%)	68	0 (0%)	24	1 (5.6%)	18
Intravitreal injection before presentation	48 (44.9%)	107	56 (75.7%)	64	21 (75%)	25	10 (55.6%)	18
Intravitreal injection at presentation	69 (53.5%)	120	44 (58.7%)	74	17 (54.8%)	28	8 (34.8%)	18
Panretinal photocoagulation before presentation	67 (55.8%)	120	48 (64.9%)	74	10 (35.7%)	28	9 (50%)	18
Panretinal photocoagulation at presentation	19 (14.7%)	120	14 (18.7%)	74	2 (7.1%)	28	3 (16.7%)	18
On no glaucoma meds at presentation	58 (48.33%)	120	41 (55.4%)	74	11 (39.3%)	28	6 (33.3%)	18
Afferent pupillary defect (APD) at presentation	9 (22%)	41	3 (15.8%)	19	4 (30.8%)	13	2 (22.2%)	9
Hyphema at presentation	20 (17%)	118	11 (14.9%)	74	5 (18.5%)	27	4 (23.5%)	17
Vitreous Heme at presentation	22 (22%)	99	16 (22.5%)	59	3 (11.1%)	25	3 (17.7%)	15
Retinal detachment at presentation	7 (7.1%)	99	7 (11.7%)	60	0 (0%)	22	2 (11.1%)	17

^a^Statistically significant age difference for PDR relative to RVO (p = < 0.001) and Other (*p* = 0.012)

Note: Continuous variables are presented with standard deviations (SD) and evaluated using the Kruskal-Wallis test. Categorical variables are presented with percentages (%) and evaluated using chi-square, exact chi-square, and the Jonckheere-Terpstra test

All patients received either an Ahmed (New World Medical, California USA) or Baerveldt (Abbot Laboratories, Illinois USA) device, or both (the analyses below that compares Ahmed to Baerveldt exclude eyes that received both). The average final IOP between the Ahmed and Baerveldt groups was not significantly different (17.0 +/− 7.6 vs 14.6+/− 7.8 respectively) and was also not significantly different when the RVO and PDR groups were analyzed separately (17.3 Ahmed vs 12.5 Baerveldt in the RVO and 17.5 vs 16.7 in the PDR). There was a statistically significant difference (*p* = 0.029) in the mean number of glaucoma medications at the final visit with Ahmed eyes (*n* = 70) requiring 1.9 medications (SD = 1.4) and Baerveldt eyes (*n* = 46) requiring 1.3 medications (SD = 1.3). There was also a statistically significant difference (*p* = 0.027) in the mean change in the number of medications from presentation to the final visit with Ahmed eyes having a mean increase of 0.6 medications (SD = 2.1) and Baerveldt eyes having a mean decrease of 0.4 medications (SD = 2.2). In subgroup analyses by etiology, this statistically significant difference in the mean change in the number of medications was found only in the RVO eyes (*p* = 0.011). There were no other significant differences between Ahmed and Baerveldt tubes for all eyes or for PDR, RVO, and Other Etiology eyes in terms of final VA, the change in VA, final IOP, the change in IOP, final number of medications, and the change in the number of medications.

**Fig. 1:**
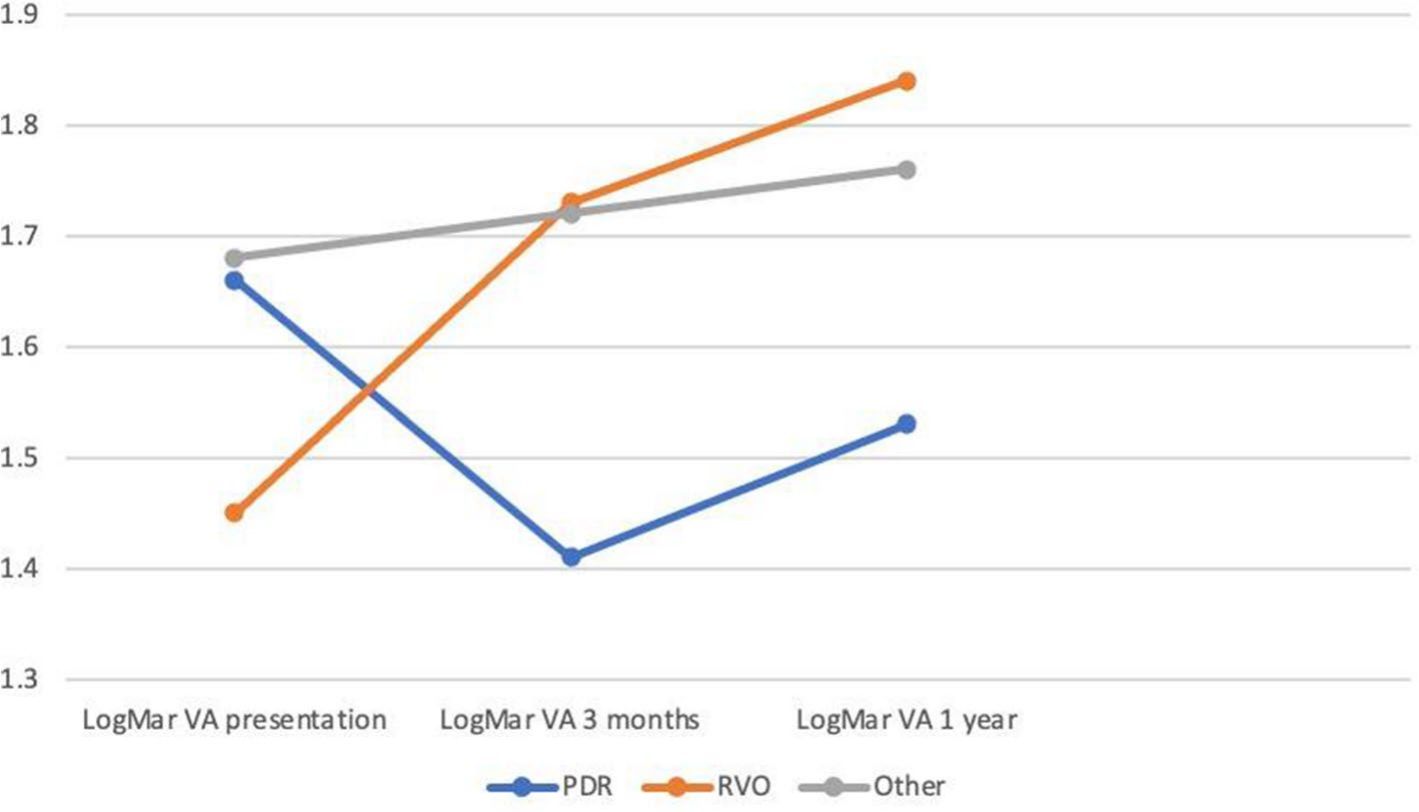
Post-operative Vision (LogMar) vs Time by etiology in patients treated with a GDD

The primary outcome measure for this study was the visual acuity at the final evaluation. The mean final LogMAR VA for the PDR group was 1.53 (20/677 Snellen) with a SD of 0.87, which was a change of + 0.13 from presentation (approximately 1 Snellen line of improvement). The mean final VA for the RVO group was 1.84 (20/1383 Snellen) with a SD of 0.65, which was a change of − 0.39 from presentation (approximately 4 Snellen lines worse). The mean final VA for the Other Etiology group was 1.76 (20/1150 Snellen) with a SD of 0.76, which represented a change of − 0.09 from presentation (approximately 1 Snellen line worse). Of note, no study eyes had a history of amblyopia. Figure 1 is a graphical representation of the VA over time for the 3 groups.

In addition to the final vision, we also examined the change in vision between groups. A VA difference of − 0.32 (approximately 20/676 for PDR and 20/1378 for RVO) at final evaluation was noted between the PDR and RVO groups in favor of PDR eyes (*p* = 0.041). Similarly, the mean VA change at final evaluation was statistically significant between PDR and RVO groups (*p* = 0.004) in favor of PDR eyes. There were no statistically significant differences in either final VA or VA change when comparing the Other Etiologies group and the PDR and RVO groups, respectively. Models were built to explore differences by etiology group in final VA and VA change at final evaluation, adjusting for whether the eye had cataract surgery (*n* = 29) or a retinal detachment (*n* = 10) between presentation and the final evaluation. Neither of the adjusting variables were statistically significant in either model, with the *p*-values for whether the eye had cataract surgery both being greater than 0.50. However, in the model for final VA differences by etiology group, adjusting for retinal detachment between presentation and the final evaluation, the *p*-value for retinal detachment = 0.0663 (marginally significant) with an adjusted final VA for eyes with a retinal detachment of 20/2641 and the adjusted final VA for eyes without a retinal detachment of 20/861. Also, in the model for VA change at final evaluation differences by etiology group, the p-value for retinal detachment = 0.0884 (marginally significant).

For all eyes, there were no significant associations between final VA and baseline IOP (*p* = 0.644) or baseline IOP groups (IOP less than 30 (*n* = 20), IOP 30–40 (*n* = 34), IOP 40–50 (*n* = 33), IOP 50+ (n = 33) [*p* = 0.923]. However, for all eyes, there were significant associations between VA change at final evaluation and baseline IOP (*p* = 0.002) and baseline IOP groups (*p* = 0.028). When IOP was considered as a continuous variable, for every 1 mmHg increase in baseline IOP, the VA change at final evaluation increased (got worse) by 0.019 logMAR units. When considering baseline IOP grouped, there were significant differences in VA change at final evaluation between IOP greater than 50 and [1] IOP less than 30 (*p* = 0.006, increase in VA of 0.721 logMAR units) and [2] IOP between 30 and 40 (*p* = 0.017, increase in VA of 0.544 logMAR units). So, having higher baseline IOP was associated with worsening VA change at final evaluation. When considering the PDR and RVO eyes individually, many of these associations were no longer significant, possibly due to reduced statistical power due to low numbers in each IOP grouping.

**Fig. 2:**
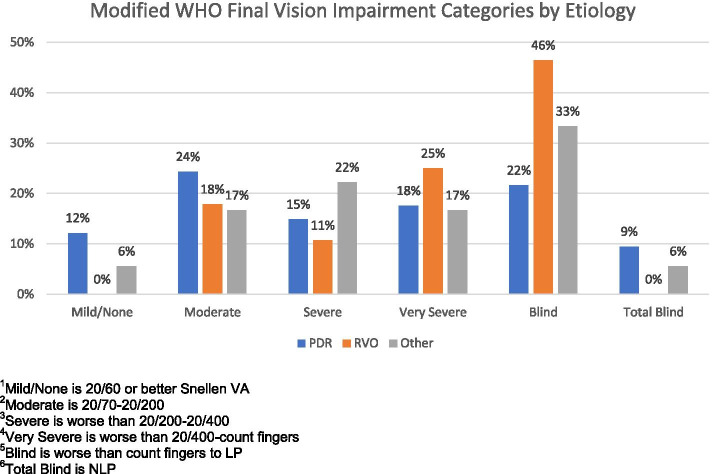
Final Vision Categories in NVG patients treated with a GDD at 1 year by etiology.^1^Mild/None is 20/60 or better Snellen VA. ^2^Moderate is 20/70–20/200. ^3^Severe is worse than 20/200–20/400. ^4^Very Severe is worse than 20/400-count fingers. ^5^Blind is worse than count fingers to LP. 6Total Blind is NLP

While it is difficult to define meaningful vision, the World Health Organization (WHO) defines vision impairment into several categories [23]. We have modified those categories to classify (by Snellen acuity) mild impairment as 20/60 or better, moderate impairment as 20/70 to 20/200, severe impairment as worse than 20/200 to 20/400 (Snellen), very severe impairment as worse than 20/400 to count fingers, blindness as hand motion to light perception, and total blindness as NLP. By these definitions, LogMar vision of 1.3 or 20/400 Snellen, or better could be considered meaningful vision. 51% percent of PDR eyes had this level of vision and 29% percent of RVO eyes had this level of vision by approximately 1 year after glaucoma surgery (*p* = 0.039). For PDR eyes, a multivariate model indicated that only VA at presentation (*p* = 0.003) and being on or starting a prostaglandin analogue (PGA) at presentation (*p* = 0.016) were independently associated with having final vision of 20/400 or better. For RVO eyes, a multivariate model indicated that only not receiving an intravitreal injection before or at presentation (*p* = 0.004) was independently associated with having this level of final vision. Figure 2 is a graphic representation of meaningful vision by etiology. Significantly more PDR.

eyes than RVO eyes had meaningful vision (mild/moderate/severe) as defined by 20/400 or better.

**Fig. 3:**
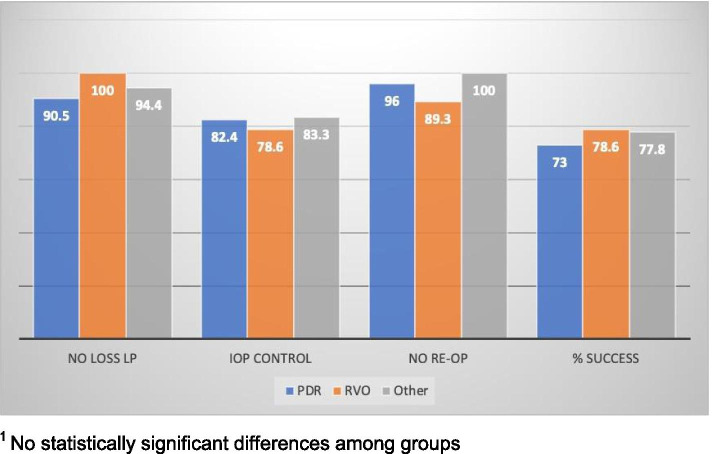
Surgical success at approximately 1 year in NVG patients treated with GDD by etiology. ^1^ No statistically significant differences among groups

The secondary outcome measure was qualified success at the final evaluation after surgery using the definitions as outlined in the methods section and similar to other prospective and retrospective analyses of GDD surgery. Overall, 90 patients (75%) had success by this definition. Regardless of etiology, most patients achieved an IOP ≤ 21 mmHg and > 5 mmHg with or without IOP lowering medications (PDR: 61/74, 82.4%; RVO: 22/28, 78.6%; Other Etiology: 15/18, 83.3%) at final evaluation. Final IOP was significantly lower for the Other Etiologies group (*n* = 18, IOP 11.9 mmHg, SD = 5.9) compared to PDR eyes (*n* = 74, 17.1 mmHg, SD = 7.9; *p* = 0.001) and RVO eyes (*n* = 28, 15.6 mmHg, SD = 7.4; *p* = 0.047). Overall medication burden on final evaluation was 1.6 (SD = 1.4) medications for all types of NVG with GDD. This was not significantly different between the groups of PDR (1.8, SD = 1.5), RVO (1.5, SD = 1.4), and Other (1.3, SD = 1.1). In total, 5% of patients across all groups required re-operation for elevated IOP. A small number of patients lost light perception vision during the study. At the final evaluation, 7 patients were NLP in the PDR group (7/74, 9.5%), 0 in the RVO group (0/28, 0%), and 1 in the Other Etiology Group (1/18, 5.6%). Of these 8 NLP patients, 3 were already NLP at POM3. IOP at presentation and POM3 did not correlate with NLP vision. It is noted that although the RVO group had worse final vision on average, none of the patients in this group lost light perception vision as opposed to 7 patients in the PDR group (9.5% vs 0%, *p* = 0.190). Figure 3 is a graphic representation of surgical success by etiology. We examined reviewed the charts of the patients who had final vision of LP and NLP at 1 year to see if there were any specific characteristics that might be evident. The clinical courses of these patients showed that some had elevated pressures in the early post-operative course, some had low pressures with choroidal effusions, some had tractional retinal detachments and some had re-operations but overall, no unifying factors were seen.

We also looked for associations between baseline characteristics and final vision of hand motion or worse vision. For the PDR eyes, having this level of final vision was associated with having worse VA at presentation (*p* = 0.023), not being on or not starting a prostaglandin analogue (PGA) at presentation (*p* < 0.001), not receiving an intravitreal anti-VEGF injection at or before presentation (*p* = 0.046), and fewer glaucoma drops initiated (or already being used) at presentation (*p* = 0.009). For the RVO eyes, having poor final vision was only associated with receiving an intravitreal anti-VEGF injection at to presentation (*p* = 0.021).

Twenty patients (16.7%) experienced surgery for a complication after GDD including tube revision, hypotony, and endophthalmitis (see Table 4). The number of complications was insufficient for statistical comparison for different types of NVG or different types of surgery.

**Table 4:** Surgical complications in NVG patients treated with a GDD

	ALL	PDR	RVO	Other	Ahmed	BGI
Re-op for low IOP (choroidal drainage and/or tube revision)	4	3 (75%)	1 (25%)	0 (0%)	0 (0%)	4 (100%)
Re-op for elevated IOP	6	3 (50%)	3 (50%)	0 (0%)	4 (66%)	2 (33%)
Suprachoroidal Hemorrhage	1	1 (100%)	0 (0%)	0 (0%)	1 (100%)	0 (0%)
Re-op for erosion	4	3 (75%)	1 (25%)	0 (0%)	1 (25%)	3 (75%)
Re-op for infection	2	2 (100%)	0 (0%)	0 (0%)	1 (50%)	1 (50%)
Retinal detachment	3	2 (66%)	0 (0%)	1 (33%)	1 (33%)	2 (66%)

A strength of this study is the relatively long follow up of patients which helped evaluate vision outcomes at approximately 1 year after surgery. As noted previously 109/120 patients had a follow up time point of approximately 1 year (all 120 eyes: mean 11.6 +/− 3.4 range 2.5–26.3 months). For those who did not have 1 year data, surrogate 3 month time point data was used (mean 3.3 +/− SD 0.9, range 2.5–4.8). We ran an analysis of the final vision with only the 109 patients that had 1 year time point (mean 12.4 +/− 2.3, range 7.4–26.3) and this did not change the statistically significant difference in final VA (*p* = 0.039) or the change in vision (*p* = 0.008) between the PDR and RVO groups.

## Discussion

This study echoed the findings of other important publications that both valved and non-valved GDDs performed similarly in the context of NVG [14, 24, 25]. Final IOP in both groups.

and across etiologies was similar with the non-valved group performing better in terms of final number of IOP lowering medications needed. From a visual acuity standpoint, PDR eyes outperformed RVO eyes at final evaluation in a clinically and statistically significant manner, again with no significant difference between valved and non-valved GDD. PDR patients receiving GDD as a group gained vision between presentation and final follow up while RVO patients lost several lines of vision. This favorable outcome held out despite more PDR eyes developing NLP vision. Although not fully characterized in the data, the reason for higher rates of NLP vision in PDR may be due to the various co-sequela that can accompany NVG in these patients (tractional retinal detachments, etc.) [26, 27]. Perhaps not surprisingly, when IOP was examined as a possible factor in determining final vision, higher baseline IOP was found to be significantly associated with the change in vision from baseline to final. When treated as a continuous variable, and also when comparing a high IOP group with a low IOP group, higher IOP was associated with larger decrease in vision at final follow up. Although there is significant literature on the relationship of elevated IOP to vision loss in NVG [1, 12, 28], we are not aware of any other studies that have shown this association specifically with GDD for NVG.

When examining functional visual outcomes, chi-square tests aimed at predicting who would have more functional vision (20/400 or better) at approximately 1 year based on presentation data yielded interesting results in PDR and RVO patients. In PDR patients, better VA at presentation was associated with having 20/400 or better final vision. It is surprising however that this was not associated with better final vision in the RVO group. This indicates that perhaps contrary to our instincts, we should not generalize potential vision for these patients if they have poor vision at presentation. The association that being on or starting a prostaglandin analog (PGA) was positive for the PDR group may indicate that more aggressive early treatment has a positive effect on final vision although unknown why the PGA in particular was beneficial and why the association was only found in the PDR group. It does seem possible that early use of PGA might have yielded better IOP control and in that way may have had the effect of better vision outcomes. There is significant literature to support the use of anti-VEGF agents in NVG for both PDR and RVO [21, 29, 30]. As such, the finding that not having a history of anti-VEGF treatment was beneficial for final vision of 20/400 or better in the RVO group may be a surrogate for those patients who had a long history of prior issues with neovascularization prior to presentation at our institution with elevated IOP.

Some of these associations held up in the analysis of eyes that had poor vision of hand motion or worse at final follow up. In the PDR group again, worse vision at presentation and not being on a PGA at presentation were associated with this level of final vision. In addition, being of fewer pressure lowering drops at presentation were also associated with poor final vision leading credibility to the argument that simply worse IOP control prior to surgery resulted in poorer final vision. In regard to anti-VEGF treatment, there were opposing findings for the PDR versus RVO group. While not having anti-VEGF at presentation in the PDR group was associated with hand motion or worse vision, in the RVO group the opposite held true. Having anti-VEGF at presentation in the RVO group was associated with poor final vision. Again, it may be that the very clinical indication of needing anti-VEGF in the RVO group may indicate a “sicker” eye that is destined to do poorly and this does not necessarily mean that we should not be doing anti-VEGF injections in our RVO patients that present with high IOP. Other studies have been published on anti-VEGF for NVG related to RVO [6], however to our knowledge, none have looked at the associated between anti-VEGF and vision outcomes in patients requiring GDD. Anti-VEGF has been found to be beneficial prior to glaucoma surgery in other studies in NVG patients [2]. This effect is likely due to its rapid onset paired with its ability to target neovascularization and vascular leakage. Instead of delaying care until the patient can be seen by a retina provider, glaucoma specialist may want to consider administering anti-VEGF themselves on presentation (with concurrent anterior chamber paracentesis). The safety profile for intravitreal injections is favorable and the skill needed to perform the procedure is relatively low with a high potential for benefit [31].

In our study, Ahmed and Baerveldt glaucoma drainage devices were the treatment of choice for NVG. Based on our results, deciding which GDD to select remains the discretion of the surgeon. If drop compliance may be an issue, selecting a Baerveldt could be advantageous as the number of drops needed at 1 year was significantly lower in our study at 1 year. This finding is consistent with the Ahmed Baerveldt Comparison study’s 3-year data [32]. The complications seen in this study were typical for GDD devices and lower than that reported in other publications (for both NVG and non-NVG patients) overall [33, 34].

Despite being one of the largest reported cohorts for NVG eyes in the literature, our study had several limitations. First, it was retrospective and therefore subject to selection and information bias. In addition, the Other Etiology group was heterogenous and the results from this group are hard to extrapolate. There were also more PDR eyes than RVO and Other Etiology eyes, which may mask differences between the groups. Finally, the providers at our tertiary referral center are also very experienced with GDD placement, which may bias results.

## Conclusions

Our data suggests patients with NVG can achieve meaningful vision after GDD for IOP control. While PDR patients did better in VA outcomes, over a quarter of RVO patients achieved VA of 20/400 or better and the majority achieved qualified success as defined by other GDD studies and none in our study were NLP at one year. Our findings suggest that there may also be a role for aggressive early use of IOP lowering medications PDR patients. Overall, the two GDD devices in our study had similar performance profiles with the non-valved device having lower average number of medications at one year follow up. Direct prospective comparison of Baerveldt, Ahmed, and cyclodestructive procedures represents a future direction for investigation of how best to treat patients presenting with elevated IOP with various etiologies of NVG.

## Data Availability

The datasets used and/or analyzed during the current study are available from the corresponding author on reasonable request.

## Abbreviations

NVG: Neovascular Glaucoma
GDD: Glaucoma Drainage Device
CPC: Cyclophotocoagulation
PDR: Proliferative Diabetic Retinopathy
RVO: Retinal Vein Occlusion
EMR: Electronic Medical Record
anti-VEGF: Anti-Vascular Endothelial Growth Factor
PRP: Pan-Retinal Photocoagulation
SD: Standard Deviation
PPV: Pars Plana Vitrectomy
PGA: Prostaglandin Analogue
CME: Cystoid Macular Edema
AGV: Ahmed Glaucoma Valve
BGI: Baerveldt Glaucoma Implant
